# Mathematical modelling of the impact of treating latent tuberculosis infection in the elderly in a city with intermediate tuberculosis burden

**DOI:** 10.1038/s41598-019-41256-4

**Published:** 2019-03-19

**Authors:** Ka Chun Chong, Chi Chiu Leung, Wing Wai Yew, Benny Chung Ying Zee, Greta Chun Huen Tam, Maggie Haitian Wang, Katherine Min Jia, Pui Hong Chung, Steven Yuk Fai Lau, Xiaoran Han, Eng Kiong Yeoh

**Affiliations:** 1 JC School of Public Health and Primary Care, The Chinese University of Hong Kong, Hong Kong, China; 2 Clinical Trials and Biostatistics Laboratory, Shenzhen Research Institute, The Chinese University of Hong Kong, Shenzhen, China; 3 Stanley Ho Centre for Emerging Infectious Diseases, The Chinese University of Hong Kong, Hong Kong, China

## Abstract

Hong Kong is a high-income city with intermediate tuberculosis (TB) burden primarily driven by endogenous reactivations. A high proportion of remote latently infected people, particularly elderly, hinders the effectiveness of current strategies focusing on passive TB detection. In this study, we developed a mathematical model to evaluate the impact of treating latent TB infection (LTBI) in the elderly in addition to current TB control strategies. The model was calibrated using the annual age-stratified TB notifications from 1965–2013 in Hong Kong. Our results showed that at present, approximately 75% of annual new notifications were from reactivations. Given the present treatment completion rate, even if only a low to moderate proportion (approximately 20% to 40%) of elderly people were screened and treated for LTBI, the overall TB incidence could be reduced by almost 50%, to reach the 2025 milestone of the global End TB Strategy. Nevertheless, due to a high risk of hepatotoxicity in elderly population, benefit-risk ratios were mostly below unity; thus, intervention programs should be carefully formulated, including prioritising LTBI treatment for high-risk elderly groups who are closely monitored for possible adverse side effects.

## Introduction

Latent tuberculosis infection (LTBI) due to *Mycobacterium tuberculosis* may persist for many years. Certain risk factors may predispose to “endogenous reactivation” of tuberculosis (TB); alternatively, “exogenous reinfection” can occur^1^.

Hong Kong is a high-income region with intermediate TB burden that has had decreasing TB notifications since the 1950s; however, this decrease has slowed since the 1970s^2–4^. Although cases resulting from primary infection and reinfection have decreased in Hong Kong, current measures using passive screening and Directed Observed Treatment, Short-course (DOTS) are insufficient as it only helps reducing recent transmissions but is unable to prevent active TB development from reactivation from remote infection, which has become the main source of TB incidence as with many intermediate burden areas^1–3^. The epidemiology of TB in Hong Kong is similar with some countries, with persistently higher incidence and risk of reactivation^2,4,5^. An early local study showed that the estimated proportion of recently transmitted TB was only 15% to 20%^6^. A study of 2243 Hong Kong residents in elderly care homes noted a high prevalence of reactivated TB^7^. Reactivation occurs predominantly in the elderly population^1,2^; thus, older-age groups account for the current TB burden, with 42.5% of notifications from people aged ≥65 years in 2016.

Under the World Health Organization (WHO) End TB Strategy, countries should target 50% and 95% reductions in the 2015 TB incidence by 2025 and 2035, respectively. To accelerate the decrease in TB incidence, targeted LTBI diagnosis and treatment have become the focus of future interventions in low-to-moderate incidence regions dominated by reactivation cases^8^. In Singapore, the TB incidence rate increased after 2008 and remained at 47/100,000 in 2017, in which an estimate of LTBI prevalence of 10.7% was found^9,10^. In Japan, the notification rates dropped slowly in recent years and an increasing number of LTBI notification from 2007 to 2016 was reported^11^. Not just in Asia, a recent investigation indicated around 1.7 billion individuals were infected with LTBI in 2014 which closed to a quarter of the world population^12^. Although United States is a low TB burden country, the elimination has not yet been met. One of the primary reasons is that the prevalence of LTBI decreased slowly between 2000 and 2012 suggesting interventions for LTBI control is needed^13^. Although LTBI diagnosis and treatment are recommended, the LTBI control varied between countries. While there is no practical programs for LTBI screening and treatment in Hong Kong, active screening of elderly in community is recommended in Japan^14^. In the United States, screening is recommended to older people being newly admitted to long-term care facilities^15^. To inform future LTBI management strategies, we investigated the public health impact of an expanded LTBI screening and treatment program targeting the elderly, as the high number of reactivated cases is a barrier for TB prevention.

LTBI treatment is available particularly for elderly with co-morbidities that imply a higher risk of progression to active diseases but acceptance and treatment completion rates vary, possibly due to adverse reactions associated with treatment, particularly hepatotoxicity induced by isoniazid (INH) therapy^16–18^.

To our knowledge, the effectiveness of LTBI treatment for elderly people in Hong Kong remains unclear. The following issues need to be addressed: while considering an active screening strategy in the elderly, would LTBI treatment alleviate a reactivation-dominated TB burden? To assess the balance between the treatment benefit and the risk from hepatitis, what are the benefit-risk ratios? To address these questions, we developed a mathematical model to evaluate the impact of LTBI treatment interventions in the elderly.

## Materials and Methods

### Data collection

Annual age-stratified TB notifications (all forms) from 1965–2013, collected from the Department of Health, were used for model fitting. Population data were collected from Census and Statistics Department reports^19,20^. Model parameter assumptions were obtained from scientific literature (Table 1). The population projection was based on the Census and Statistics Department statistical report^21^. The ethics approval was obtained from The Joint Chinese University of Hong Kong – New Territories East Cluster Clinical Research Ethics Committee. Data were retrospectively collected in aggregate form and publicly available so no informed consent was required. All methods were performed in accordance with the relevant guidelines and regulations.

**Table 1:** Summary of parameters used in the models.

Parameter	Definition	Assumptions	Sensitivity analysis assumptions
*N* ^*t*^_*a*_	Population size of a-th age group at time *t*	Varied by years^19^	—
*δ* ^*t*^	Number of new births at time *t*	Varied by years^20^	—
*μ* ^*t*^_*a*_	Net rate of natural mortality and migration of a-th age group at time *t*	Varied by years^4,20^. The ratio of mortality between older to younger groups were assumed at 7:1, 9:1, 12:1, 16:1, and 20:1 for year <1981, 1981–1990, 1991–2000, 2001–2010, and >2010 respectively.	—
*φ* ^*t*^	BCG vaccination proportion on new births at time *t*	Varied by years^50^	Uniform (99%, 99.9%)
*ρ*	BCG efficacy	74%, an estimate based on 4 randomized-controlled trials^26^	Uniform (62%, 83%)^26^
*λ* ^*t*^_*a*_	Force of infection for age group *a* at time *t*	—	—
*β*_*ak*_	Age-specified transmission rate from age group *k*-th to *a*-th	Estimated by MCMC using the TB notification data^50^	Follow posterior distributions
*τ*	Transition rate from youngers to elders	—	—
*α*	Proportion developing recent latent infection stage from remote latent and recovered stages	0.013^3^	Uniform (0.011, 0.015)^3^
*υ*_*s*_	Progression rate from recent latent infection to remote latent	0.2 per year	—
*υ*_*i*_	Progression rate from recent latent infection to infectious or non-infectious stage	0.03 per year^22,51,52^	Uniform (0.02, 0.04)^22,51,52^
*σ*_*a*_	Progression rate from remote latent infection to infectious or non-infectious stage	0.0005 per year for younger adults. A multiplier *K* to this value was assumed for elders and estimated by the MCMC approach^2,22,51,52^	Follow the posterior distribution
*γ*	Proportion of infectious TB from either fast or slow latent TB	0.85^3^	Uniform (0.75, 0.95)^3^
*q*	Proportion of infectious and non-infectious individuals being detected and treated	0.9^3,53–55^	Uniform (0.80, 0.99)^3,53–55^
*p*	Proportion of treated individuals able to complete TB treatments	0.25 for years before 1970; 0.6 for years 1970–1978; 0.87 after year 1978^53–55^	Uniform (0.80, 0.95)
*κ*_*c*_	Efficacy for treatment completion	0.93^53–55^	Uniform(0.85, 0.99)
*κ*_*d*_	Efficacy for treatment defaulted	0.84^53–55^	Uniform(0.65, 0.90)
*ξ*	Progression rate from treatment to recovered stage	Reciprocal for 6 months	—
*ϕ*	Natural annual recovery rate	0.2^3,22,51,52^	Uniform (0.1, 0.3)^3,22,51,52^
*ω*	Relapse rate	0.045^50^	Uniform (0.017, 0.17)^2,22,50^
*m*	Screened proportion (acceptability) of LTBI in elders	Test for different proportions	—
*r*	Detection rate of LTBI	77%, an average sensitivity of the tuberculin skin test^16^. Enhancing sensitivity of 90% (T-spot TB test) was also tested.	—
*h* ^*j*^	LTBI treatment adherence level for duration *j*	Duration *j* can be 52, 24, and 12 weeks; the current adherence levels were respectively assumed to be 65% (complete treatment), 15%, and 5%^16,29,30^. We varied the treatment completion rate from 0–80% to assess the changes (24- and 12-weeks completion rate was unchanged).	
*η* ^*j*^	Progression rate from LTBI treatment with duration *j* to recovered stage	Reciprocal for 52, 24, and 12 weeks of isoniazid therapy^16,27,28^	—
*κ*_*l*_^*j*^	Efficacy for different durations of LTBI treatment	75%, 65%, and 21% for 52, 24, and 12 weeks of treatments, respectively^17,29^	—

### Disease transmission model

**Figure 1:**
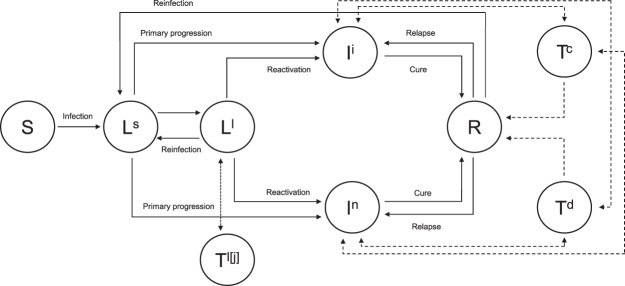
Schematic flow of the age-stratified compartmental model for TB transmissions. TB, tuberculosis; S, susceptible; L^s^, latently infected (recent); L^l^, latently infected (remote); I^i^, infectious; I^n^, non-infectious; R, recovered; T^c^, treatment completion; T^d^, treatment defaulted; T^l[j]^, LTBI treatment for duration *j*.

We extended the age-structured compartmental model to study TB dynamics and the impact of interventions^2,3,22,23^. Compartments were built based on transmission characteristics. Essential features of the model are shown in Fig. 1. The differential equations describe the transition process of number of individuals between classes given different rates of changes. For model equations, the rate of change in a state is denoted by an upper dot (·) which is the first derivative with respect to time. We stratified the individuals into two age groups: below 60 years old (youngers, *a* = 1) and greater than or equal to 60 years old (elders, *a* = 2). For example, ${\dot{S}}_{2}$ represents the rate of change in the number of susceptibles in the elderly. The age groups are discretized given a younger-to-elder transition rate *τ*.

Denoting the current number of individuals among a population, who are susceptibles (*S*_*a*_), recent latent TB infections (*L*^*s*^_*a*_), remote latent TB infections (*L*^*l*^_*a*_), infectious (*I*^*i*^_*a*_), non-infectious (*I*^*n*^_*a*_), successfully treated (*T*^*c*^_*a*_), treatment defaulted (*T*^*d*^_*a*_), receive LTBI treatments adhering with duration *j* (*T*^*l[j]*^_*a*_), and recovered (*R*_*a*_) in *a*-th age group, the model equations are described as follows:

#### Susceptible

According to population dynamics, susceptible individuals were defined as those without TB infection who were not vaccinated or had vaccine failure. The number of susceptibles increased with the number of new birth (*δ*^*t*^) by time *t*. As BCG vaccination was given to newborns and school children since early 1950s, a proportion of them is immunized at a rate *φ*^*t*^ at time *t* given a vaccine efficacy *ρ*. They are infected with an age-specified force of infection *λ*^*t*^_*a*_. We assumed only sputum smear-positive individuals can transmit TB in the transmission equations. Net rate of natural mortality and migration in *a*-th age group (*μ*^*t*^_*a*_) is included in all compartments. The discretized differential equations read:

$${\dot{S}}_{1}=(1-\rho {\phi }^{t}){\delta }^{t}-({\lambda }_{1}^{t}+\tau +{\mu }_{1}^{t}){S}_{1}$$

$${\dot{S}}_{2}=\tau {S}_{1}-({\lambda }_{2}^{t}+{\mu }_{2}^{t}){S}_{2}$$

and

$${\lambda }_{1}^{t}=\sum _{k=1}^{2}{\beta }_{1k}\frac{{I}_{k}^{i}}{{N}_{k}^{t}},{\lambda }_{2}^{t}=\sum _{k=1}^{2}{\beta }_{2k}\frac{{I}_{k}^{i}}{{N}_{k}^{t}},$$

where *β*_*ak*_ is the age-specified transmission rate from age group *k*-th to *a*-th and *N*^*t*^_*k*_ is the population size of age group *k*-th at time *t*. Because of different contact patterns among youngers and elders, the *β*_*ak*_ forms the “who acquires infection from whom” (WAIFW) mixing matrix^24^:

$${\beta }^{M}=(\begin{array}{cc}{\beta }_{11} & {\beta }_{12}\\ {\beta }_{21} & {\beta }_{22}\end{array})$$

#### Latently infected (recent)

Infected individuals move into the recent latent class. Recovered and remote latent TB subjects can also be re-infected at a rate *α* and move to this class. The recent latent subjects can develop active disease at a rate *υ*_*i*_ or progress to remote latent class at a rate *υ*_*s*_.

$${{\dot{L}}^{s}}_{1}={{\lambda }^{t}}_{1}{S}_{1}+\alpha {{\lambda }^{t}}_{1}({R}_{1}+{{L}^{l}}_{1})-({\upsilon }_{s}+{\upsilon }_{i}+\tau +{{\mu }^{t}}_{1}){{L}^{s}}_{1}$$

$${{\dot{L}}^{s}}_{2}=\tau {{L}^{s}}_{1}+{{\lambda }^{t}}_{2}{S}_{2}+\alpha {{\lambda }^{t}}_{2}({R}_{2}+{{L}^{l}}_{2})-({\upsilon }_{s}+{\upsilon }_{i}+{{\mu }^{t}}_{2}){{L}^{s}}_{2}$$

#### Latently infected (remote)

Individuals who are not active TB ≤ 5 years of the recent latent period move to the remote latent stage^2,23^. We defined TB reactivation as a TB infection that developed ≥ 5 years after initial infection. A constant reactivation rate was assumed, and we estimated a fractional increase (*K*) in progression rate from elderly-young individuals. Those progressed to remote latent can develop active disease in either infectious or non-infectious at a rate *σ*_*a*_. As for intervention testing, a proportion *m* of the latent subjects in elderly are screened at a sensitivity *r* and treated with LTBI treatment with adherence level of duration *js* i.e. 52 (whole course), 24, and 12 weeks. A number of treated LTBIs returns to this compartment given a proportion of LTBI treatment failure (1- *κ*_*l*_^*j*^) due to limited efficacy levels of different durations.

$${{\dot{L}}^{l}}_{1}={\upsilon }_{s}{{L}^{s}}_{1}-(\alpha {{\lambda }^{t}}_{1}+{\sigma }_{1}+\tau +{{\mu }^{t}}_{1}){{L}^{l}}_{1}$$

$${{\dot{L}}^{l}}_{2}=\tau {{L}^{l}}_{1}+{\upsilon }_{s}{{L}^{s}}_{2}+{\sum }_{j}{\eta }^{j}(1-{{\kappa }_{l}}^{j}){{T}^{l[j]}}_{2}-(\alpha {{\lambda }^{t}}_{2}+{\sigma }_{2}+{\sum }_{j}{h}^{j}mr+{{\mu }^{t}}_{2}){{L}^{l}}_{2}$$

#### Infectious and non-infectious

Given that infectious TB cases are predominantly sputum smear-positive (microscopic examination), these subjects are defined as being infectious with a proportion *γ*. Those smear negative or extrapulmonary TB cases are defined as being non-infectious. Primary progression to active TB can occur ≤ 5 years of the first infection or result from reinfection from recovered and remote latent individuals i.e. the infectious and non-infectious individuals are progressed from recent and remote latently infected. They can be detected and treated with a proportion *q*, and the remaining individuals (1- *q*) will be naturally cured at a rate *ϕ*. Given limited efficacy for completed treatment (1- *κ*_*c*_) and defaulted treatment (1- *κ*_*d*_), treated individuals can progress to these compartments at a rate *ξ*. Recovered individuals can also relapse and move into these classes.

$${{\dot{I}}^{i}}_{1}=\gamma [\omega {R}_{1}+{\upsilon }_{i}{{L}^{s}}_{1}+{\sigma }_{1}{{L}^{l}}_{1}+(1-{\kappa }_{c})\xi {{T}^{c}}_{1}+(1-{\kappa }_{d})\xi {{T}^{d}}_{1}]-[q+\tau +(1-q)\varphi +{{\mu }^{t}}_{1}]{{I}^{i}}_{1}$$

$${{\dot{I}}^{i}}_{2}=\tau {{I}^{i}}_{1}+\gamma [\omega {R}_{2}+{\upsilon }_{i}{{L}^{s}}_{2}+{\sigma }_{2}{{L}^{l}}_{2}+(1-{\kappa }_{c})\xi {{T}^{c}}_{2}+(1-{\kappa }_{d})\xi {{T}^{d}}_{2}]-[q+(1-q)\varphi +{{\mu }^{t}}_{2}]{{I}^{i}}_{2}$$

and

$${{\dot{I}}^{n}}_{1}=(1-\gamma )[\omega {R}_{1}+{\upsilon }_{i}{{L}^{s}}_{1}+{\sigma }_{1}{{L}^{l}}_{1}+(1-{\kappa }_{c})\xi {{T}^{c}}_{1}+(1-{\kappa }_{d})\xi {{T}^{d}}_{1}]-[q+\tau +(1-q)\varphi +{{\mu }^{t}}_{1}]{{I}^{n}}_{1}$$

$${{\dot{I}}^{n}}_{2}=\tau {{I}^{n}}_{1}+(1-\gamma )[\omega {R}_{2}+{\upsilon }_{i}{{L}^{s}}_{2}+{\sigma }_{2}{{L}^{l}}_{2}+(1-{\kappa }_{c})\xi {{T}^{c}}_{2}+(1-{\kappa }_{d})\xi {{T}^{d}}_{2}]-[q+(1-q)\varphi +{{\mu }^{t}}_{2}]{{I}^{n}}_{2}$$

#### Treatment completion and defaulted

After passive screening, infectious and non-infectious individuals are treated with anti-TB drugs and they can complete treatment (at least 6 months) or default treatment respectively with proportions *p* and (1-*p*). The individuals can recover or be failure to treatment at a rate *ξ*.

$${{\dot{T}}^{c}}_{1}=pq({{I}^{i}}_{1}+{{I}^{n}}_{1})-(\xi +\tau +{{\mu }^{t}}_{1}){{T}^{c}}_{1}$$

$${{\dot{T}}^{c}}_{2}=\tau {{T}^{c}}_{1}+pq({{I}^{i}}_{2}+{{I}^{n}}_{2})-(\xi +{{\mu }^{t}}_{2}){{T}^{c}}_{2}$$

and

$${{\dot{T}}^{d}}_{1}=(1-p)q({{I}^{i}}_{1}+{{I}^{n}}_{1})-(\xi +\tau +{{\mu }^{t}}_{1}){{T}^{d}}_{1}$$

$${{\dot{T}}^{d}}_{2}=\tau {{T}^{d}}_{1}+(1-p)q({{I}^{i}}_{2}+{{I}^{n}}_{2})-(\xi +{{\mu }^{t}}_{2}){{T}^{d}}_{2}$$

#### LTBI treatment

The screened proportion (*m*) of remote latent subjects are treated with LTBI therapy with an efficacy *κ*_*l*_^*j*^ and a recovery rate *η*^*j*^ corresponding to different durations. The *h*^*j*^s are the tested proportions of subjects in different adherence levels for duration *j*. Subjects with different adherence are stratified into *j* compartments i.e. *T*^*l[j]*^.

$${\dot{T}}^{l{[j]}_{1}}={h}^{j}mr{{L}^{l}}_{1}-({\eta }^{j}{{\kappa }_{l}}^{j}+\tau +{{\mu }^{t}}_{1}){T}^{l{[j]}_{1}}$$

$${\dot{T}}^{l{[j]}_{2}}=\tau T{l}^{{[j]}_{1}}+{h}^{j}mr{{L}^{l}}_{2}-({\eta }^{j}{{\kappa }_{l}}^{j}+{{\mu }^{t}}_{2}){T}^{l{[j]}_{2}}$$

#### Recovered

Infected subjects can be cured naturally or by treatments and they can move into the recovered class. Recovered individuals can relapse at a rate *ω* to either infectious or non-infectious. They can also be re-infected to the latent stage at a rate *α*.

$${\dot{R}}_{1}=(1-q)\varphi ({{I}^{i}}_{1}+{{I}^{n}}_{1})+\xi ({\kappa }_{c}{{T}^{c}}_{1}+{\kappa }_{d}{{T}^{d}}_{1})-(\alpha {\lambda }_{1}+\omega +\tau +{{\mu }^{t}}_{1}){R}_{1}$$

$${\dot{R}}_{2}=\tau {R}_{1}+(1-q)\varphi ({{I}^{i}}_{2}+{{I}^{n}}_{2})+\xi ({\kappa }_{c}{{T}^{c}}_{2}+{\kappa }_{d}{{T}^{d}}_{2})-(\alpha {\lambda }_{2}+\omega +{{\mu }^{t}}_{2}){R}_{2}$$

Natural deaths were considered in all compartments. We assumed no imported cases in the model setting, as migrant infections comprise a low proportion of TB in Hong Kong.

### Model calibration for baseline scenario

In the initial stages of model calibration, we assumed all of the population in 1965 born before 1952 (age >13 years) were susceptible or infected with TB i.e. ~60% of total population as BCG vaccination has been given to newborns since 1952^19,20^. Following Wu *et al*.^3^, we assumed 70% prevalence of LTBI with 20% recent latent infections. The initial numbers of active TB cases were assumed at 2.5 times the number of TB notifications.

The baseline scenario was calibrated by fitting annual age-stratified TB notification data to the model. The first 5 years of data (1965–1969) were discarded from model fitting to remove the burn-in period of the model simulations. Fixed parameters are summarised in Table 1. The model generated age-stratified number of TB new cases (for infectious and non-infectious) was fitted to the observed annual notifications by using the Markov Chain Monte Carlo (MCMC) method in a Bayesian inferential framework. A likelihood function was formed by assuming Poisson-distributed TB notifications:

$$L({\rm{\Theta }})={\prod }_{t=1}\frac{{e}^{-{\eta }_{t}}{{\eta }_{t}}^{{x}_{t}}}{{x}_{t}!}$$

where *x*_*t*_ and *η*_*t*_ are respectively the observed and the expected new cases generated by the transmission model at time *t*. In the MCMC estimation, flat prior distributions were assumed for the parameters (Θ) i.e. *β*_*11*_, *β*_*12*_, *β*_*21*_, *β*_*22*_, and *K*. A random walk Metropolis algorithm was used to obtain the posterior distributions. Totally 10,000 iterations were used as the burn-in period and the subsequent 100,000 iterations were used to draw the posterior estimates. Step sizes were selected to obtain acceptance proportions between 20% and 40%. The convergence of the MCMC chains was diagnosed with time-series trace plots and tested by Gelman and Rubin convergence measure^25^. Auto-correlation of the MCMC chains was checked by auto-correlation function plots.

Assuming the Bacillus Calmette–Guérin (BCG) vaccination was implemented at a constant rate of 99.4% (average over past 5 years) and no LTBI was treated, a forecast of TB incidence (number of cases/total population size per 100,000) to year 2030 was simulated for the baseline scenario. The variation in births, mortality, and net movement was in accordance with projected statistics.

To assess the goodness of fit for the model calibration, R-square, mean absolute error (MAE), and mean absolute percentage error (MAPE) were used. In addition, a retrospective validation process using data from 1965–2003 for model fitting and data from 2004–2013 for validation was conducted to examine the consistency in terms of predictive power.

The deterministic equations of the mathematical model and calibration were programmed using R software version 3.0.3 (R Foundation for Statistical Computing, Vienna, Austria).

### Scenarios for active screening and treatment of TB in the elderly

In Hong Kong, diagnosis of LTBI is commonly established using the tuberculin skin test (TST), followed by 6–12 months treatment with INH offered to all patients. The T-Spot. TB test is an alternative method for LTBI diagnosis, but is not currently in the local strategic protocol^16^. A shorter course of weekly INH and rifapentine regimen is sometimes offered to elderly patients with a recent study indicated it is as effective as INH treatment alone^26^. Given the scenario that LTBIs in the elderly group were screened annually and those found positive were treated, we examined the impact of varying proportions of LTBI patients who were willing to be screened (acceptability) and who completed treatment (adherence) on the annual TB incidence starting from 2014. Additional compartments (T^l[j]^) were added to the baseline model for LTBI patients who adhered to the *j*-th treatment duration: 52 (whole course), 24, and 12 weeks (Fig. 1). The diagnostic sensitivity for LTBIs in the elderly was fixed at 76% from TST and 93% from T-spot. TB test^17^. The current practice for the duration of INH therapy in Hong Kong was fixed at 12 months although WHO recommended 6 months duration of INH is adequate treatment for LTBI^27,28^. Adherence levels affected treatment effectiveness in the elderly population. Present adherence levels were assumed to be 65%, 15%, and 5% for 52, 24, and 12 weeks’ treatment, respectively^16,29,30^, with corresponding efficacies of 75%, 65%, and 21%, respectively^16,29^. The averted incidence (i.e. reduction of TB incidence when the LTBI intervention was applied, compared with no intervention scenario in a year) was determined according to differing acceptability and treatment completion rates.

As hepatitis is a major safety indicator of INH treatment, the benefit-risk ratio between the number of active TB cases prevented and the number of hepatitis events by year was determined^16,29^. The number of hepatitis events was calculated as the product between the number of cases treated and the elderly hepatitis rates assumed to be 2.3%, 2.0%, and 1.7% for 52, 24, and 12 weeks of INH treatment, respectively^16,29,31^.

### Sensitivity analysis

A sensitivity analysis was conducted by obtaining the partial rank correlation coefficients (PRCCs) between the parameter values and the averted TB incidence to explore the sensitivity of the selected model parameters^32,33^. Plausible distributions of fixed parameters (i.e. BCG vaccination rate, BCG efficacy, proportion developing recent latent infection stage, progression rates from recent and remote latent infection to infectious or non-infectious stage, proportion of infectious TB, proportion of infectious and non-infectious individuals being detected and treated, proportion of treated individuals able to complete TB treatments, efficacies for treatment completion or treatment defaulted, natural recovery rate, and relapse rate) were assumed given plausible ranges of values (Table 1). Fitted parameters were assumed to follow their posterior distributions.

In addition, multivariate sensitivity analysis was conducted to assess the impact of varying all selected parameters on the uncertainty of the annual TB incidence. The 95% credible intervals (CI) of the simulation outcomes were drawn over 10,000 realisations.

## Results

### Baseline scenario

**Figure 2:**
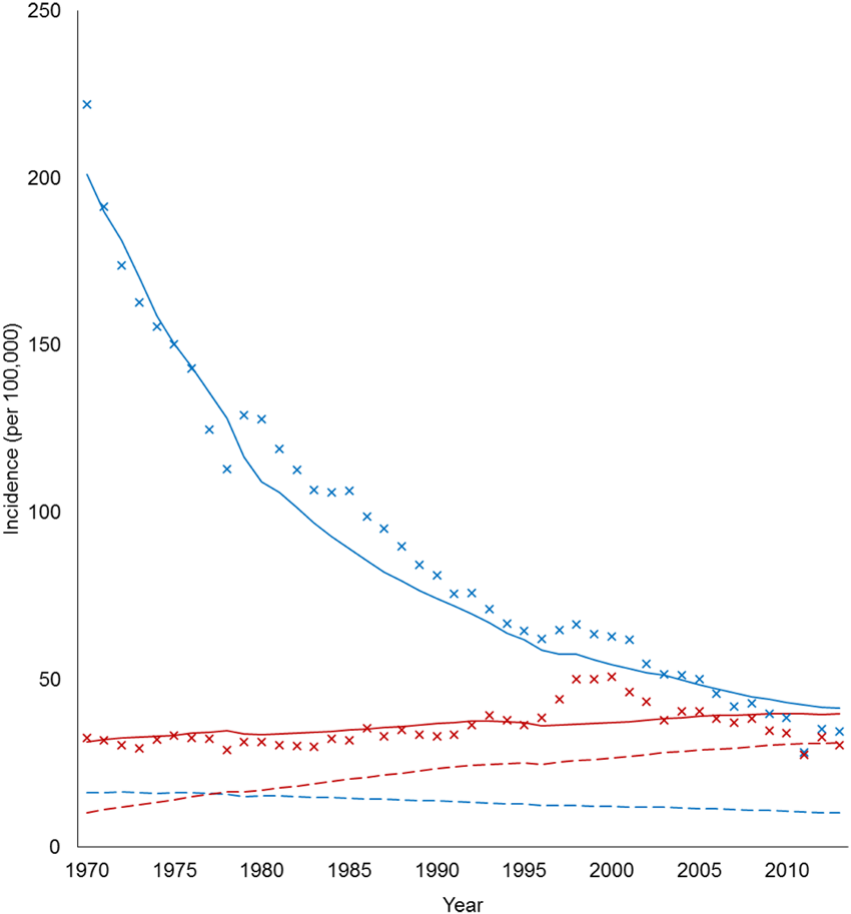
Observed (cross) and fitted (solid line) TB incidence by age <60 (blue) and ≥60 years (red). Reactivated cases are presented in dash lines. TB incidence was calculated by dividing the number of cases by total population per 100,000. TB, tuberculosis.

Figure 2 shows the observed and fitted TB incidence by age and estimated number of reactivated cases. All MCMC chains of the estimated parameters were well-converged given the Gelman and Rubin convergence measure approach to unity. Posterior distributions of the model parameters are shown in Supplementary Information File. The R-square, MAE, and MAPE of the model fitness were 96.4%, 9.6/100,000, and 8.8% respectively showing a high level of agreement between observed data and model simulated TB infection trend. In the retrospective validation using 2004–2013 data, we showed that the R-square, MAE, and MAPE of the model simulation were 89.5%, 6.8/100,000, and 8.2% respectively which indicated a high consistency in model prediction.

In the baseline scenario, TB incidence in younger individuals gradually decreased from 222/100,000 in 1970 to 38/100,000 in 2010, while incidence in elderly patients remained at ~30–40/100,000 throughout the study period (Fig. 2). Reactivated cases in the elderly steadily increased from 8/100,000 in 1970 to 30/100,000 in 2010. In the elderly, the proportion of reactivations increased from 31% in 1970 to almost 75% in 2010. From the estimates, the reactivation rate was 3 times higher in elderly patients than young patients (*K* = 3.33).

### LTBI interventions

**Figure 3:**
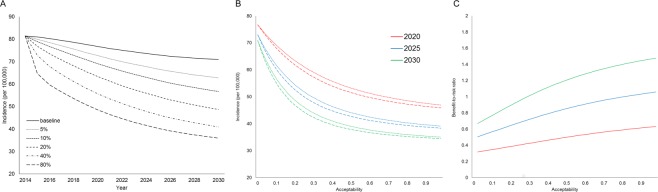
(**A**) Annual TB incidence by year among different acceptability levels in the elderly with LTBIs. (**B**) Annual TB incidence at year 2020 (red), 2025 (blue), and 2030 (green) according to acceptability levels. TST (dots line) and T-spot (dash lines) were used for LTBI screening. (**C**) Benefit-risk ratios at year 2020 (red), 2025 (blue), and 2030 (green) according to acceptability levels. The Benefit-risk ratios were obtained by dividing the number of active cases prevented with the number of hepatitis cases that occurred in the specified year. TB, tuberculosis; TST, tuberculin skin test.

Figure 3 shows the impact of LTBI interventions on changes in annual TB incidence by year, according to various acceptability rates treatment in the elderly, considering the present level of treatment adherence (65% completion rate). If no elderly cases were treated, TB cases decreased slowly from 2014–2030 with ~10/100,000 incidence reduction. However, if a small number of elderly LTBI cases were screened, i.e. 10% annually after 2014, the reduction rate could be similar in 2022, with a 15/100,000 averted incidence in 2030. A 20% treatment acceptability could induce a 20/100,000 decrease in incidence from 2014–2021, while a 40% acceptability could reduce the annual incidence by almost 50% in 2025 (~40/100,000). Increasing the acceptability for LTBI screening to 80% resulted in a substantial decrease in incidence (>20/100,000), but did not generate a large difference in later years, i.e. ~5/100,000 reduction in cases from 2025–2030.

LTBI treatment did not substantially impact the overall control when acceptability was set at >40% (Fig. 3B). The difference in TB incidence between 2025 and 2030 was only ~6/100,000 with a treatment acceptability between 40–80%. If the LTBI detection rate was increased to 93% (e.g. using T-spot. TB), the additional effect on incidence reduction was minimal (Fig. 3B).

**Figure 4:**
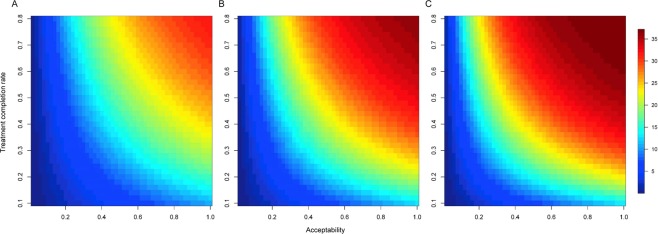
Averted TB incidence at year 2020 (**A**), 2025 (**B**), and 2030 (**C**) according to the varied treatment completion rates and acceptability in the elderly with LTBI. LTBI, latent tuberculosis infection; TB, tuberculosis.

Figure 4 shows the averted incidence in 2020, 2025, and 2030 with varying acceptability and LTBI treatment completion rates. In general, maintaining the treatment completion rate at >60% and increasing acceptability resulted in observable effects on the incidence averted. Compared with the no intervention scenario, if the treatment completion rate was enhanced to 80%, a 40% acceptability obtained ~20 and 30/100,000 averted incidence in 2020 and 2030, respectively. Nevertheless, despite maintaining a high treatment completion rate, a higher acceptability (>40%) could not draw observable long-term impact. An 80% acceptability could yield ~30/100,000 cases averted in 2025, but only yield 4/100,000 additional cases averted in 2030. Meanwhile, the LTBI intervention became less effective for incidence reduction when the treatment completion rate was low. When the treatment completion rate was as low as 20%, increasing the acceptability to 40% in the elderly yielded only ~12/100,000 averted incidence in 2030.

### Benefit-risk ratio

Given a relatively high hepatitis rate, benefit-risk ratios were kept at a low level when the present level of treatment adherence was maintained (Fig. 3C). Even if the LTBI intervention was given annually with high acceptability, benefit-risk ratios were below unity before 2025, i.e. the number of active TB cases prevented were fewer than the number of hepatitis events. However, preventing a TB case reduced further transmission chains, and the intervention could obtain a benefit-risk ratio greater than unity in 2030, given >30% acceptability.

**Figure 5:**
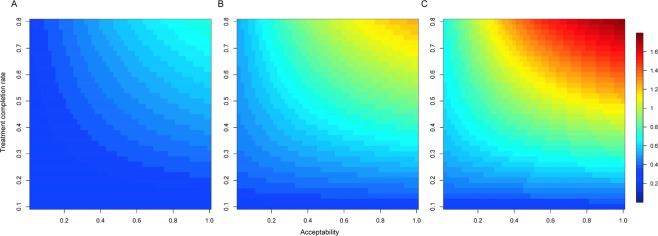
Benefit-risk ratios at year 2020 (**A**), 2025 (**B**), and 2030 (**C**) according to the varied treatment completion rates and acceptability in the elderly with LTBI. LTBI, latent tuberculosis infection.

Benefit-risk ratios remained at low levels and did not vary substantially (range, 0.2–0.7) in the early years with varying acceptability and treatment completion rates (Fig. 5). Meanwhile, if current treatment completion rate of the annual LTBI intervention was maintained or increased to >65%, a remarkable gain in benefit-risk ratios was observed in later years, even with moderate acceptability (Fig. 5B,C).

### Sensitivity analysis

**Figure 6:**
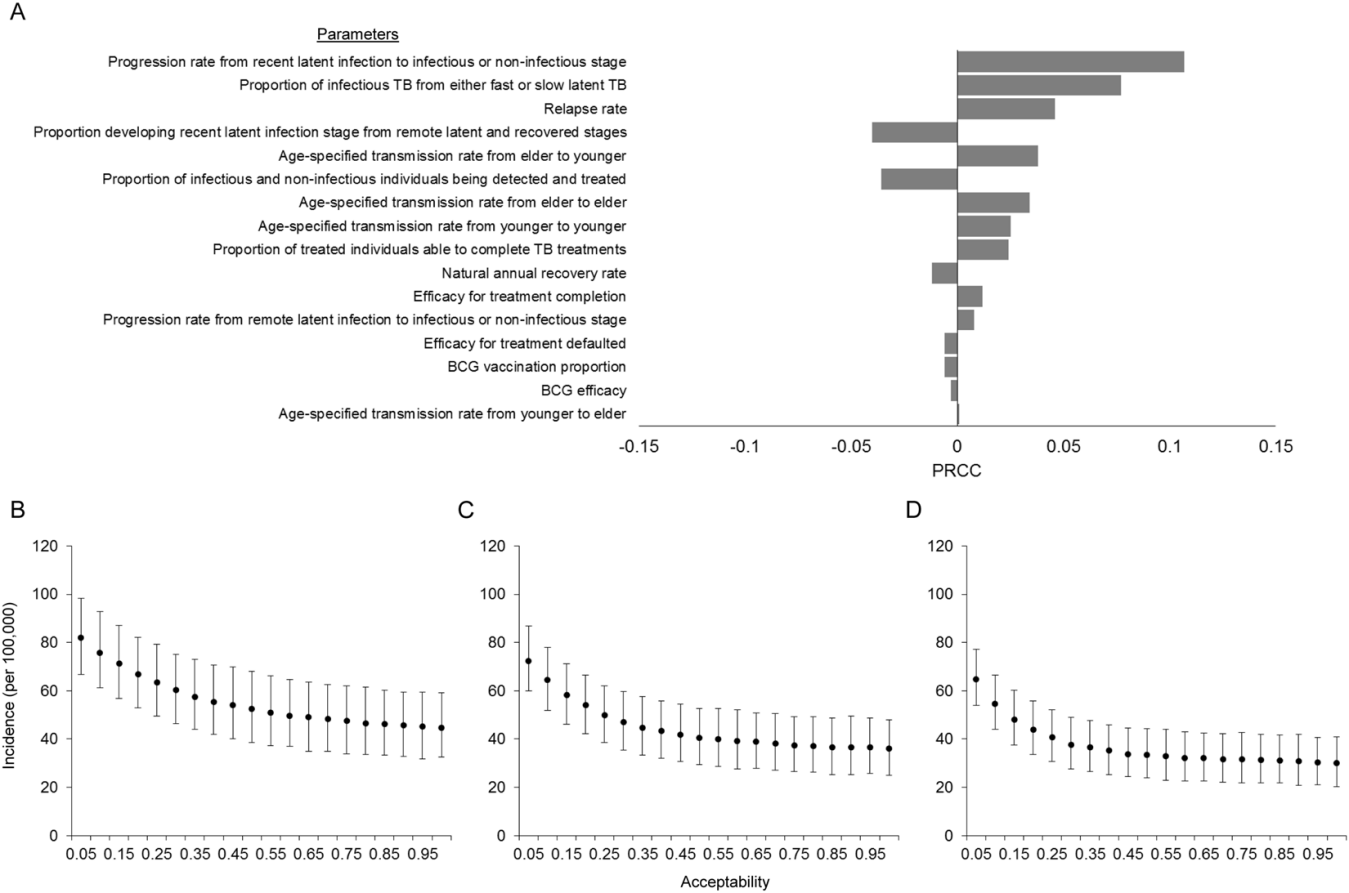
Sensitivity analysis: (**A**) PRCCs of the parameters and multivariate-simulated TB incidence in year 2020 (**A**), 2025 (**B**), and 2030 (**C**). PRCCs, Partial rank correlation coefficients; TB, tuberculosis.

A sensitivity analysis was conducted to explore the sensitivity of the model parameters and the tornado diagram showed that the simulated averted incidences were not sensitive when the parameters varied (Fig. 6A). Among the parameters, the progression rate from recent latent infection and proportion of infectious TB had the most influence on the simulated averted incidence (PRCC = 0.107 and 0.077, respectively) as the effect of the TB treatment largely relied on the generated number of infectious cases based on these rate parameters. The absolute numbers of the PRCCs from all the other parameters were all <0.05.

Figure 6B,C shows the simulated scenarios for changes in TB incidence when model parameters varied multivariately. In general, the overall trend of the impact from LTBI interventions was consistent with the abovementioned results. The error intervals of the 95% CIs were approximately ±15, ±12, and ±10/100,000 for the TB incidence in 2020, 2025, and 2030, respectively.

## Discussion

The high number of LTBI cases in elderly individuals and the associated risk of reactivation poses a challenge to TB control, with management in this population likely to improve control. Particularly in some regions with low to moderate TB burden, slow decline in TB has been attributed to the cases that develop through reactivation. With a high reactivation rate occurring in the elderly population, a timely evaluation of the effectiveness of LTBI treatment for elderly people is able to offer a valuable advice for TB elimination. In this study, we assessed the impact of LTBI screening and treatment varying the acceptability and adherence from elderly people. Our results showed that at present, reactivations comprise approximately 75% of all TB cases. Given the present treatment completion rate, if ~20–40% of elderly people were willing to be screened and treated for LTBI, the overall TB incidence could be reduced by almost 50% long-term. If the treatment completion rate can be maintained at >65%, increasing acceptability could substantially impact the averted incidence. Nevertheless, this effect tended to taper off in later years.

The number of active TB cases prevented were fewer than the number of hepatitis events that occurred in the early years when the LTBI intervention was provided annually, due to hepatotoxicity. Despite high acceptability and treatment adherence, the intervention did not prevent >2 active cases per hepatitis event long-term. As few diagnostic and treatment guidelines targeting TB in the elderly have been established^2,34^, our study profiles the effectiveness of LTBI interventions in the elderly, and our findings can be used when developing strategies optimised for LTBI screening and treatment.

Similar to other studies^2,3^, our results showed that TB incidence, comprised primarily of elderly reactivation cases, cannot be adequately controlled with current strategies using passive case finding and DOTS. Corroborating an earlier study in Hong Kong^3^, we showed that approximately 75% of new TB cases were from reactivations, consistent with findings from China^35^. The TB burden in Japan is similar to Hong Kong, in which 71.8% of new cases in 2016 were attributable to people aged >60 years, who likely had reactivated infection^36^. In contrast to Hong Kong, the Japanese government has targeted groups at high risk of developing TB in the LTBI treatment program, provided that side-effects and other drug effects were carefully monitored in the elderly receiving the chemoprophylaxis^37^.

To investigate the impact of LTBI measures, Huynh *et al*.^35^ developed an individual-based model to investigate whether TB interventions could help achieve the WHO’s End-TB target in China. It was reported that preventative therapy in elderly patients who were screened and treated appropriately for LTBI could help reduce the TB incidence to 60% in 2025. Although the TB epidemiology in China is different from that in Hong Kong, their findings are comparable with those obtained in our study. Although we showed that TB incidence can be substantially reduced long-term, the current strategy for detection and treatment of LTBI will not achieve the End-TB target of 90% reduction or maintenance of ~10/100,000 in Hong Kong by 2035^8^, the current level for low-burden countries^38^.

In general, only a low proportion of patients with LTBIs develop disease. In this study, we found that benefit-risk ratios remained at low levels when the elderly population were screened and treated for LTBI. This indicates that offering LTBI treatments to a general elderly population should be carefully considered. However, LTBI screening and treatments should be prioritized to specific high-risk groups^3^. WHO defines high-risk groups as individuals with particular pathological conditions, those who are in contact with active TB patients, and those in high-risk living or work settings^39^. In Hong Kong, a study showed 68.6% of the elderly home residents were positive with the TST screening results^7^. The estimated LTBI prevalence was comparatively higher than an earlier local study and that in other countries^40^. Despite these results, the prevalence of hepatotoxicity could be greater among local elderly patients owing to the high prevalence of chronic hepatitis B carriers in Hong Kong^7^. The optimal approach for delivering interventions, likely to involve stratifying patients according to hepatotoxicity risk and vigilant monitoring during treatment, would require prudent evaluations.

Apart from drug toxicity, we showed that poor adherence to LTBI treatment can adversely impact the reduction of TB incidence. We showed that the present LTBI treatment completion rate was able to achieve a desirable averted incidence, and this appears comparable to the 75% rate obtained in a similar setting that offered self-administered INH over 6 months in Australia^41^. Investigations have shown that the elderly were even more likely to adhere to INH treatment than younger patients^42,43^. In addition, the estimates in the study offer a valuable reference for the number of LTBIs needed to be approach in achieving a greater benefit with required level of acceptance and tolerance as they can vary by regions and populations according to the Public Health England^44^. Apart from that, the study findings may give additional values for implying a revision of related policy. For example, a recent published clinical trial showed that 4-month of rifampin was not inferior to the typical 9-month of INH for the prevention of active TB and it was associated with a higher rate of treatment completion (i.e. ~15%) and reduction of hepatotoxic events^45^. Based on our estimates, such increase in treatment completion would yield >30/100,000 cases averted and a benefit-risk ratio greater than unity in 2025 even with a low acceptability in the elderly population.

The current study has several major limitations. In the mathematical model, we did not capture demographic and epidemiological characteristics such as sex, TB-associated mortality, and drug-resistant TB. While we did not modulate TB-associated mortality as an outcome in the simulations, local studies reported that approximately 4% mortality is currently associated with a new TB notification^34^. Using this figure, >100 deaths could be prevented in 2030 if 20% of elderly patients with LTBI were detected and treated. Moreover, we acknowledged simply using hepatitis events to determine a benefit-risk ratio may not be comprehensive enough when comparing with typical benefit-risk analysis as there are other potential side effects from the treatment (e.g. rash, nausea, malaise, and fever), even though it has been employed in different investigations^16,29^. For example, Sadatsafavi *et al*. (2013) conducted a throughout cost-benefit analysis and the risks including all adverse events, TB reactivation, and deaths were quantified in terms of quality-adjusted life-years^46^. However, we tend to use a simple benefit-risk ratio to study a trade-off between effectiveness and safety of an intervention program within the scope of our study. Also, our modelling results could be overestimated, as a proportion of drug-resistant TB cases might be included in the analyses. In Hong Kong, the prevalence of INH- and/or rifampicin-resistant TB is low; the rates for mono-drug-, multi-drug-, and extensively drug-resistant TB were 7.97% (325 cases), 0.834% (34 cases), and 0.0491% (2 cases) respectively^47^. No evidence has suggested that prophylactic treatment for LTBI causes drug-resistant bacilli strains^48^, and drug resistance was more likely due to transmission of resistant strains. In Hong Kong, household transmissions of MDR-TB were found, but were rare^49^.

In conclusion, this study showed that for a city with intermediate TB burden primarily driven by reactivations, if LTBI interventions can be provided to the elderly and maintained at the current treatment completion rate of 65%, treating a low-moderate proportion of individuals with LTBIs annually will reduce the TB incidence by 50%, to reach the 2025 milestone of the global End-TB Strategy. Nevertheless, due to a high risk of hepatotoxicity in the elderly population, benefit-risk ratios were comparatively low; thus, intervention programs for optimising screening and treatment strategies for LTBI should be carefully considered. Our findings underscore the need for new anti-TB drugs/drug regimens with greater efficacy and safety for treating LTBI.

## Supplementary information

Supplementary Information File
