# Supplementary material for: Mathematical modelling of the impact of treating latent tuberculosis infection in the elderly in a city with intermediate tuberculosis burden
Source: Sci Rep. 2019 Mar 19;9:4869. doi: 10.1038/s41598-019-41256-4 (PMC6424958; doi:10.1038/s41598-019-41256-4)
Supplement: Supplementary file 1 — Supplementary Information File [file 41598_2019_41256_MOESM1_ESM.pdf]

Mathematical modelling of the impact of treating latent tuberculosis infection in the elderly in a city with intermediate tuberculosis burden

Authors and affiliations:

Ka Chun Chong [a,b*], Chi Chiu Leung [c], Wing Wai Yew [a], Benny Chung Ying Zee [a,b], Greta Chun Huen Tam [a], Maggie Haitian Wang [a,b], Katherine Min Jia [a], Pui Hong Chung [a], Steven Yuk Fai Lau [a], Xiaoran Han [a], and Eng Kiong Yeoh [a*]

[a] JC School of Public Health and Primary Care, The Chinese University of Hong Kong, Hong Kong, China

[b] Clinical Trials and Biostatistics Laboratory, Shenzhen Research Institute, The Chinese University of Hong Kong, China

[c] Tuberculosis and Chest Service, Department of Health, Hong Kong, China.

* **Corresponding author**

Eng Kiong Yeoh

Mailing address: Rm 501, School of Public Health and Primary Care, The Chinese University of Hong Kong, Hong Kong, China

Telephone number: 852 22528716

E-mail: yeoh_ek@cuhk.edu.hk

Supplementary Information File: Supplementary figure S1

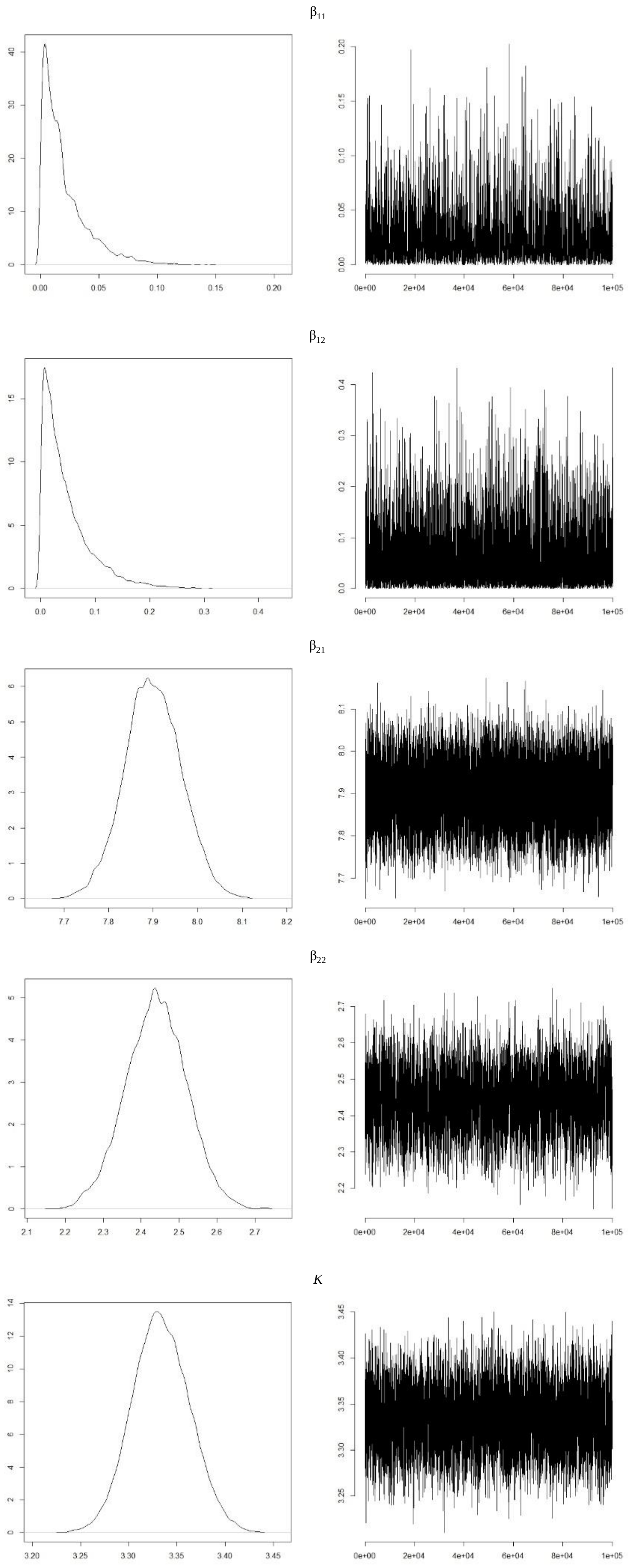

Figure S1. MCMC density functions (left panel) and convergence plots (right panel) for the posterior distributions of estimated parameters
